# D-Transposition of Great Arteries With Infective Endocarditis and Congestive Cardiac Failure: A Case Report

**DOI:** 10.7759/cureus.56832

**Published:** 2024-03-24

**Authors:** Chandrakant M Bokade, Nisha R Aglave, Pooja B Nagrale

**Affiliations:** 1 Pediatrics, Datta Meghe Medical College, Datta Meghe Institute of Higher Education & Research, Nagpur, IND

**Keywords:** arterial switch operation, balloon atrial septostomy, congestive cardiac failure, infective endocarditis, transposition of great arteries, cyanotic congenital heart disease, rastelli procedure

## Abstract

Embryological misalignment between the aorta and pulmonary trunk gives rise to the congenital anomaly of the heart known as transposition of the great arteries (TGA). TGA is a type of parallel circulation, where the heart pumps oxygenated blood from the left ventricle into the pulmonary trunk. The deoxygenated blood from the right ventricle is circulated into the body as it pumps blood into the aorta. This type of parallel circulation is not compatible with life unless there is communication between oxygenated and deoxygenated blood. The presence of a ventricular septal defect (VSD) or patent ductus arteriosus (PDA) in TGA patients serves as this communication. Cyanosis in the first month of life is the most common presenting feature. We had a five-and-a-half-year-old male child presenting with cyanosis and congestive cardiac failure (CCF), along with infective endocarditis with mitral valve regurgitation, which is an unusual complication of dextro-TGA (d-TGA) with pulmonary stenosis (PS) with VSD.

## Introduction

Approximately 4.7 per 10,000 live births have transposition of the great arteries (TGA), which is the second most common cause of cyanotic heart disease, with the first being tetralogy of Fallot (TOF). Three percent of all congenital heart diseases and 20% of cyanotic heart diseases are TGA. It is the most frequent cyanotic congenital heart disease (CCHD) manifesting in the first week after birth [1]. There is transposition or switching of the position of two main arteries carrying blood out of the heart: the main pulmonary artery and the aorta in TGA, called ventriculoarterial discordance [2]. The solitus relationship is present when the aorta is placed to the right of the pulmonary artery and posteriorly. In contrast, in around 60% of cases with dextro-TGA (d-TGA), the aorta is positioned to the right of the pulmonary artery but anteriorly. Conversely, in a subset of patients with levo-TGA (l-TGA), the aorta may be placed anteriorly and to the left of the pulmonary artery.

The cardiac abnormalities linked to TGA are ventricular septal abnormalities, blockage of the left ventricular outflow tract, anomalies of the tricuspid and mitral valves, and variations in the coronary arteries. They present with cyanosis, tachypnoea, and murmur, resulting from the associated cardiac defects. Fetal echocardiography can rarely diagnose TGA. If clinical features are suggestive of cyanotic heart disease, then chest radiography, echocardiography, and, rarely, cardiac catheterization would be helpful for diagnosis [3]. The initial management includes infusion of prostaglandin E1 and performing balloon atrial septostomy. Following the achievement of hemodynamic stability, corrective surgery can be undertaken. Definitive surgical procedures, such as the arterial switch operation (ASO) and Rastelli procedure, should be conducted within the first week of life [4].

## Case presentation

A male child, aged five and a half, born of non-consanguineous marriage, first by order, presented with fever, cough, and cold persisting for 12 days. Difficulty in breathing has been noted for the past 10 days, increasing in severity from the New York Heart Association (NYHA) grades one to four over the last two days. The child developed edema starting from the feet and progressing to anasarca.

The baby, born full-term through normal vaginal delivery with a birth weight of 3,200 g, did not require NICU admission. The mother observed a bluish discoloration of the baby's body on the 12th day of life, leading to investigations and a diagnosis of congenital cyanotic heart disease. The child had no past hospitalization history for lower respiratory support, congestive cardiac failure, or thromboembolic phenomena. There was minimal cyanosis and no history of hypercyanotic spells. A general examination revealed the child to be febrile, with a heart rate of 114 per minute, respiratory rate of 56 per minute, nasal flaring, and respiratory distress. Blood pressure in all four limbs ranged between the 50th and 90th percentiles. The child exhibited central cyanosis involving the lips, tongue, palms, and soles, with an oxygen saturation of 62% in room air and grade III clubbing. Signs of polycythemia, such as prominent veins over the face, chest, and abdomen, flushed face, and conjunctival congestion were present. Continuous high-grade fever was noted, but no clinical signs of infective endocarditis were observed. Edema was widespread (pedal, sacral, ascites, facial puffiness), and the jugular venous pressure (JVP) was elevated at 9 cm. No jaundice or facial dysmorphism was noted, and there were no external congenital anomalies. The child was severely underweight and stunted, with a weight of 14 kg (< 3rd percentile, Indian Academy of Pediatrics (IAP) growth charts), height of 103 cm (< 3rd percentile, IAP growth charts), and a body mass index (BMI) of 13.2 kg per meter square (underweight). Cardiovascular examination revealed a left precordial bulge, epigastric pulsations, and an apical impulse shifted to the lateral and down to the left midclavicular line. Prominent veins were visible over the chest wall and abdomen. The apical impulse was hyperdynamic and palpable in the left fifth intercostal space one cm lateral to the midclavicular line, with a systolic thrill over the second left intercostal space, left sternal border, and apical area. A grade III intensity parasternal heave was present. Auscultation revealed a soft first heart sound in the mitral area, along with a holosystolic murmur of grade IV best heard over the left third and fourth intercostal spaces, transmitted to the left axilla and parasternal area, and best heard with the diaphragm of the stethoscope. In the tricuspid area, a holosystolic murmur of grade IV was heard. In the aortic area, A2 was audible; conversely, in the pulmonary area, S2 was single. No ejection click was noted. A continuous murmur was heard in the interscapular, infrascapular, and infra-axillary regions, grade III, best heard with the diaphragm of the stethoscope, suggestive of collaterals.

Respiratory system examination revealed coarse basal crepitations bilaterally. A continuous murmur of collaterals was heard over the interscapular, infrascapular, and infra-axillary areas. Abdominal examination showed ascites with shifting dullness. Hepatomegaly was present with a liver span of 13 cm. The spleen was not palpable. The clinical diagnosis of congenital cyanotic heart disease with decreased pulmonary blood flow, most likely Fallot's physiology, was made. Differential diagnosis included double outlet right ventricle, tricuspid atresia, transposition of the great vessels with pulmonary stenosis (PS), and total anomalous pulmonary venous return. Blood investigations revealed polycythemia with raised hematocrit value (Table 1).

**Table 1:** Blood investigations on admission

Blood investigation	Observed value	Age-related reference range
Hemoglobin (Hb)	14.8 g/dL	11.5–14.2 gram/dL
Hematocrit (Hct)	62%	35–55%
White blood cells (WBC)	7,800/cubic mm	5,000–19,000/cubic mm
Platelet counts	240,000/cubic mm	150,000-450,000/cubic mm

**Figure 1:**
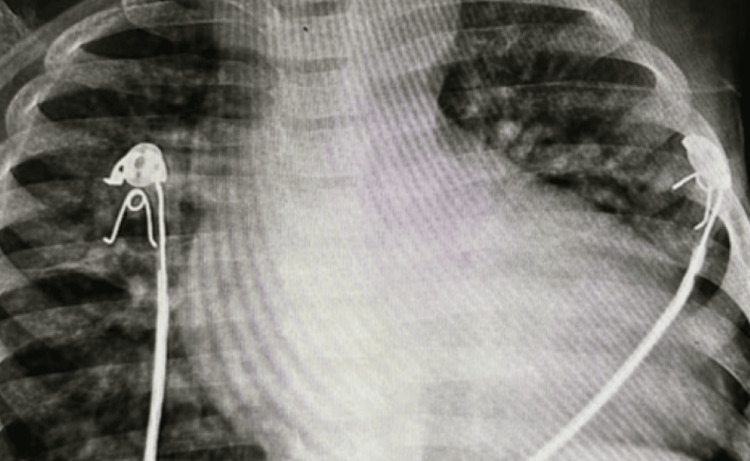
Chest X-ray PA view showing minimally oligemic lung field with the cardiac shadow showing an “egg on string” appearance PA: Posteroanterior

The chest X-ray (CXR) indicated a minimally oligemic lung field with the cardiac shadow showing an “egg on string” appearance (Figure 1).

**Figure 2:**
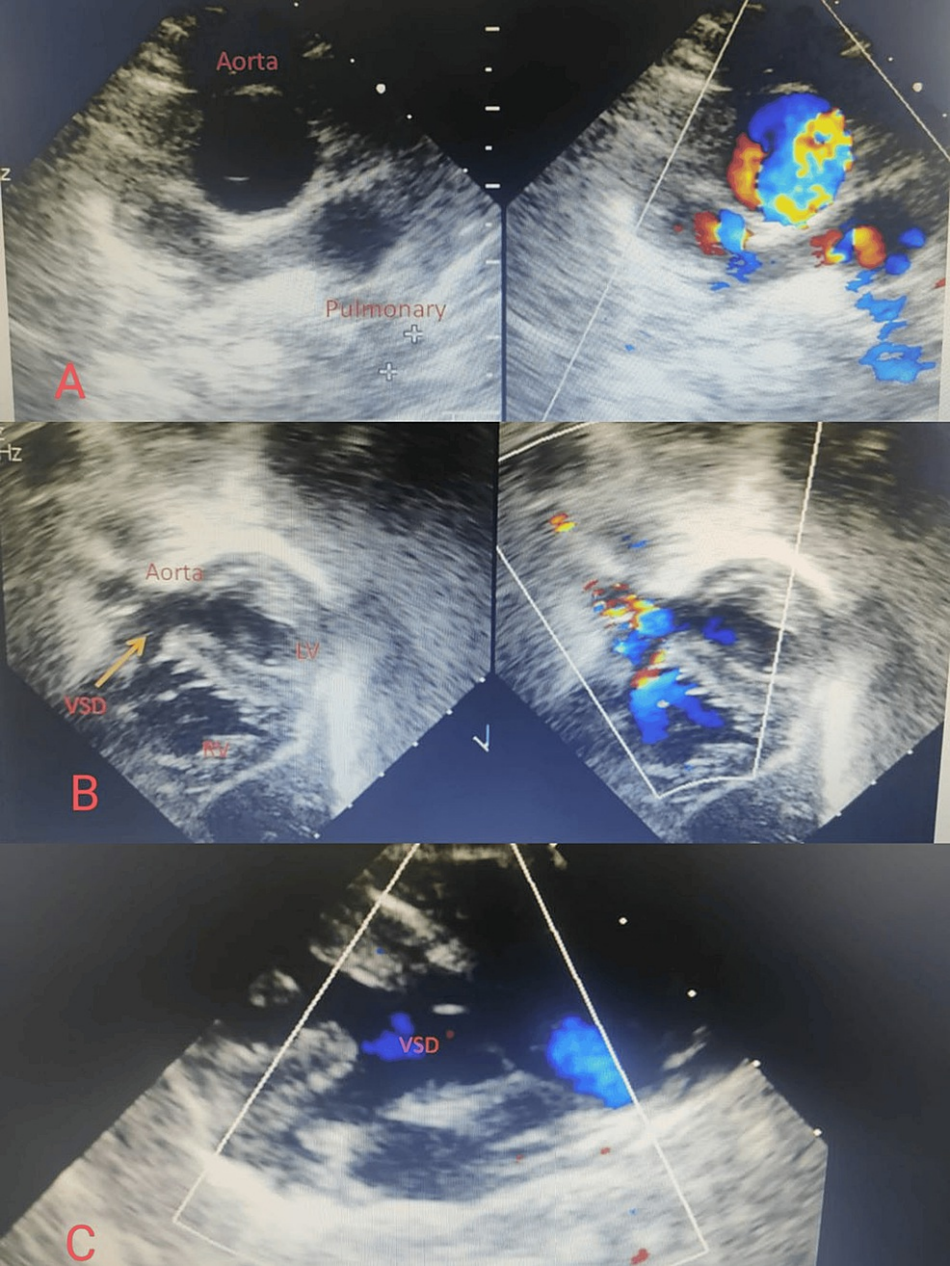
Two-dimensional echocardiography Image A: Showing great artery relationship with the aorta being anterior and to the right suggestive of transposition of great arteries, Image B: Subcostal view showing two good-sized ventricles and large VSDs and pulmonary outflow tract. Image C: Parasternal view showing large subpulmonic VSD with override VSD: Ventricular septal defect, LV: Left ventricle, RV: Right ventricle

The initial two-dimensional echocardiography suggested d-TGA (d-TGA), bidirectional VSD, moderate PS, and severe mitral regurgitation (MR). Despite starting empirical antibiotics, the patient's fever did not subside. Suspecting infective endocarditis, echocardiography was repeated after 72 hours of admission, revealing vegetations over the mitral valve, measuring 3.5 x 4 mm over the anterior mitral leaflet (AML), 3 mm x 2.5 mm over the posterior mitral leaflet (PML), and 2.5 x 2 mm over the subpulmonic area, with severe MR. There was a prolapse of the anterior mitral valve leaflet, which was the reason for severe MR (Figure 2).

Oxygen therapy with a non-rebreather mask at 8 L/minute was initiated. Decongestive measures, including injection furosemide, restricted fluids, and oral enalapril were started. Injectable antibiotics, specifically injection of piperacillin-tazobactam at 100 mg/kg/dose every eight hours and injection of vancomycin at 15 mg/kg/dose every six hours, were administered while awaiting the blood culture reports. Because of no response to the initial treatment after seven days with persistent fever, the growth of Pseudomonas, and an increase in the size of vegetations on repeat two-dimensional echocardiography, higher antibiotics injection colistin 5 mg/kg/day divided every six hours and injection of ceftazidime 100 mg/kg/day divided every eight hours were initiated based on the culture sensitivity report.

A cardiologist was consulted who advised to continue decongestive treatment and antibiotics for six weeks. The patient was planned for palliative surgery, followed by corrective surgery after the resolution of infective endocarditis. Unfortunately, the child did not show signs of recovery even after seven days of antibiotic therapy, and there was persistent high-grade fever. On the 22nd day of admission, the child experienced a deterioration in health, transitioning into septic shock accompanied by pneumonia and multiorgan failure. Despite all the efforts made, the child could not be revived.

## Discussion

In the pediatric population, infective endocarditis is an infrequent but severe condition linked to significant morbidity and mortality. Recent shifts in the epidemiology of infective endocarditis among children reveal CHD as the predominant predisposing factor in developed regions, whereas rheumatic heart disease has decreased in frequency [5]. The parallel circulation between pulmonary and systemic circulation in TGA is incompatible with extrauterine life. The survival after birth depends upon the prostaglandin infusion and balloon atrial septostomy started immediately as soon as suspicion of TGA was made. Moreover, it depends on the degree of mixing of oxygenated with deoxygenated blood. Our patient had one admission at day of life 12 for cyanosis, but later he did not have hypercyanotic spells because he had developed multiple collaterals that served as feeding vessels for pulmonary circulation. The blood from vessels supplying the pulmonary arteries would be oxygenated and carried the oxygenated blood to the systemic circulation; hence, this child did not have hypercyanotic spells. Computed tomography angiography (CTA) was indicated for better visualization of collaterals but could not be due to financial reasons. 

Infective endocarditis in unrepaired TGA is a rare presentation. Infective endocarditis prophylaxis is indicated in presurgical patients and only for six months after the surgery [6]. In the absence of a particular focus, Staphylococcus aureus, Viridans streptococci, Streptococcus gallolyticus, and Haemophilus species, Actinomycetemcomitans, Cardiobacterium hominis, Ekinella corodens, Kingellaspecies (HACEK), or community-acquired enterococci are typical bacteria for infective endocarditis from two distinct blood cultures [7]. This child had a growth of atypical organism Pseudomonas aeruginosa in blood culture, which is a rare cause of infective endocarditis. Pseudomonas-causing infective endocarditis is commonly observed in patients with intravenous drug users and those with prosthetic values. We were unable to identify the source of the Pseudomonas infection, but the sensitivity pattern was different from regular Pseudomonas isolates from the hospital. Because of an atypical organism causing infective endocarditis, although appropriate treatment was started, the child went into septic shock. Mitral regurgitation resulting from valvular vegetation and valve prolapse is a recognized complication of infective endocarditis. In this case, the patient presented with severe mitral valve regurgitation, leading to congestive cardiac failure as the primary cause. Managing d-TGA poses challenges. The key approach involves promptly initiating prostaglandin E1 infusion and performing balloon atrial septostomy in a cyanotic neonate, followed by definitive surgical intervention as the cornerstone of treatment. Infective endocarditis and its complications are observed in 5.0%-6.7% of children with congenital heart disease. Notably, infective endocarditis in CCHD exhibits a mortality rate 3.6 times higher than that in non-CCHD [8]. Patients with complex congenital heart diseases need to undergo various procedures such as conduit procedures, palliative shunts, patches, and prosthetic valves. The risk of infective endocarditis is on the rise in patients with or without such a procedure. Advances in surgery have significantly enhanced survival rates, exceeding 90% [9]. Long-term survival and functional results are best achieved by the arterial switch operation (ASO). After discharge, several studies show a more than 95% rate of survival after 15-25 years [10].

## Conclusions

We presented a five-and-a-half-year-old male child with unrepaired d-TGA, ventricular septal defect (bidirectional), moderate PS, and severe MR, experiencing an atypical complication of infective endocarditis with congestive cardiac failure. Atypical organisms should be suspected in patients not responding to the usual treatment for infective endocarditis. The presence of collateral and severe MR is a cause of congestive cardiac failure in association with infective endocarditis. Emphasizing surgical repair in early life improves survival rate.
